# Nesprin-1-alpha2 associates with kinesin at myotube outer nuclear membranes, but is restricted to neuromuscular junction nuclei in adult muscle

**DOI:** 10.1038/s41598-019-50728-6

**Published:** 2019-10-02

**Authors:** Ian Holt, Heidi R. Fuller, Le Thanh Lam, Caroline A. Sewry, Sally L. Shirran, Qiuping Zhang, Catherine M. Shanahan, Glenn E. Morris

**Affiliations:** 1 Wolfson Centre for Inherited Neuromuscular Disease, RJAH Orthopaedic Hospital, Oswestry, SY10 7AG UK; 2 Institute for Science and Technology in Medicine, Keele University, Keele, ST5 5BG UK; 3 BSRC Mass Spectrometry and Proteomics Facility, Biomedical Sciences Research Complex, University of St Andrews, North Haugh, St Andrews, Fife KY16 9ST UK; 4 Cardiovascular Division, James Black Centre, King’s College, London, SE5 9NU UK

**Keywords:** Nuclear envelope, Differentiation, Nuclear envelope, Neuromuscular disease

## Abstract

Nesprins, nuclear envelope spectrin-repeat proteins encoded by the SYNE1 and SYNE2 genes, are involved in localization of nuclei. The short isoform, nesprin-1-alpha2, is required for relocation of the microtubule organizer function from centromeres to the nuclear rim during myogenesis. Using specific antibodies, we now show that both nesprin-1-alpha2 and nesprin-1-giant co-localize with kinesin at the junctions of concatenated nuclei and at the outer poles of nuclear chains in human skeletal myotubes. In adult muscle, nesprin-1-alpha2 was found, together with kinesin, only on nuclei associated with neuromuscular junctions, whereas all adult cardiomyocyte nuclei expressed nesprin-1-alpha2. In a proteomics study, kinesin heavy and light chains were the only significant proteins in myotube extracts pulled down by nesprin-1-alpha2, but not by a mutant lacking the highly-conserved STAR domain (18 amino-acids, including the LEWD motif). The results support a function for nesprin-1-alpha2 in the specific localization of skeletal muscle nuclei mediated by kinesins and suggest that its primary role is at the outer nuclear membrane.

## Introduction

The SYNE1^1^ (or MYNE1^2^) gene was first identified from the expression of the 111 kDa protein now known as nesprin-1-alpha2 which is expressed almost exclusively in skeletal muscle and heart^3^. This was quickly found to be a short isoform of a more widely-expressed, full-length 1008 kDa product of the SYNE1 gene^4^ now known as nesprin-1-giant, and also known as enaptin^5^. Nesprin-1-giant is largely an alpha-helical rod structure consisting of 74 spectrin repeats^6^. The amino-terminus of nesprin-1-giant has calponin homology domains that bind the actin cytoskeleton while its carboxy-terminus contains transmembrane and KASH (Klarsicht-ANC-Syne-homology) domains which localize it to the outer nuclear membrane, with the KASH domain extending into the lumen of the nuclear envelope^6^. SUN (Sad1/UNC-84) domain proteins form trimers and span the inner nuclear membrane, with their amino-terminal nucleoplasmic domains interacting with lamin A/C in the nuclear lamina and their carboxyl-terminal “SUN” domains interacting with KASH domains of nesprins inside the lumen, thereby forming a LINC (linker of nucleoskeleton and cytoskeleton) complex^7,8^.

Nesprin-1-alpha2 (112 kDa) has a unique 31 amino acid sequence at its amino-terminus, but is otherwise identical in sequence to the carboxy-terminus of nesprin-1-giant^4,6^. Nesprin-1-alpha2 contains 6 spectrin repeats, equivalent to repeats 69 to 74 of nesprin-1-giant. Between spectrin repeats 71 and 72 there is an unstructured and highly conserved 18 amino acid sequence called the STAR domain^6^. The STAR domain includes a four amino-acid LEWD motif which binds kinesin^9^ and this in turn mediates interaction with microtubular motor systems. Another highly-conserved 23 amino acid sequence lies 114 nucleotides downstream of the STAR domain and is encoded by an alternatively spliced exon (Delta-SR or DV23^3,6^) with greater than 95% inclusion in cardiac and skeletal muscle^3^; its function is unknown. The majority of the mutations in nesprins that cause Emery-Dreifuss muscular dystrophy or an inherited cardiomyopathy are located in the region of SYNE1 gene that encodes nesprin-1-alpha2^10,11^.

The closely-related SYNE2 gene^6^ produces a 792 kDa nesprin-2-giant and a 61 kDa muscle-specific form, nesprin-2-alpha1, though the form with a structure most similar to nesprin-1-alpha2 is the 103 kDa nesprin-2-epsilon2^6^, which is expressed in heart and other tissues, but not in skeletal muscle^3^, while the 122 kDa nesprin-2-epsilon-1 is expressed during very early development^3,12^.

The fusion of mononucleated myoblasts gives rise to multinucleated myotubes that mature into the contractile myofibres with peripheral nuclei of adult skeletal muscle. The movement and positioning of nuclei are essential steps in muscle development, steps that require nesprin-1-alpha2^13^ and nesprin-2^14,15^. With the fusion of myoblasts, nuclei are initially found at the centre of the myotube. The nuclei are then re-located along the length of the myotube and later, *in vivo*, move to the periphery to anchor at the sarcolemma with a few clustering at neuromuscular junctions^16^. Nesprin-1-alpha2 is required for the localization of mAKAP (A-kinase anchoring protein 6) to the outer nuclear membrane via the third spectrin repeat of mAKAP^17^. Nesprin-1-alpha2 is also involved in the localization of centrosomal proteins, such as PCM-1^18^ and A-kinase anchoring protein 9 (AKAP9, also known as AKAP450)^19^, to the nuclear envelope of myotubes, AKAP9 being required for microtubule nucleation and nuclear spreading during differentiation, independently of kinesin^19^. Nesprin-2 levels are higher than nesprin-1 in mature adult muscle, while nesprin-1 is higher in immature and regenerating fibres with central nuclei^20^, consistent with a special function of nesprin-1 during early development. The movement of nuclei away from the centre to the periphery of myofibres, which occurs later in development, may require nesprin-2 again^14,15,21,22^. In drosophila muscle, klarsicht (nesprin) is required for the correct assembly of sarcomeres by ZASP, which is located at the outer nuclear membrane of these pre-positioned nuclei within the myofibre^23^.

Most antibodies that recognize nesprin-1-alpha2 also recognize nesprin-1-giant, but in the present study, we have used monoclonal antibodies specific for either nesprin-1-alpha2 or nesprin-1-giant^24^ to show that both nesprin-1 isoforms, unlike nesprin-2, concentrate at the junctions of concatenated nuclei and at the outer poles of the nuclear chains in human skeletal myotubes, together with kinesin. In adult skeletal muscle, nesprin-1-alpha2 was restricted to the special nuclei associated with neuromuscular junctions (NMJ), with kinesin still present, while nesprin-1-giant is present on most nuclear membranes. We have further shown by proteomic analysis that kinesin heavy- and light-chains are the only major interaction partners of the STAR domain (LEWD motif) and we were unable to detect interactions of KASH-less nesprin-1-alpha2 with either emerin or lamin A/C. The results are consistent with functions at the outer nuclear membrane for all nesprin isoforms with a KASH domain and we re-evaluate the evidence for the popular hypothesis that nesprin-1-alpha2 has additional functions at the inner nuclear membrane or lamina^25^.

## Results

### Kinesin heavy and light chains are the only proteins pulled down by intact nesprin-1-alpha2 but not by nesprin-1-alpha2 lacking the STAR domain which contains the LEWD motif

Site-directed mutagenesis was used to produce nesprin-1-alpha2 lacking the 18-amino-acid STAR domain (STARdel) as a bacterial recombinant protein with a GST tag at the C-terminus (Supplementary Fig. S1). Successful deletion was confirmed by DNA sequencing (Supplementary Fig. S2). Both STARdel and wild-type (WT) nesprin-1-alpha2 were attached to glutathione beads and used to pull-down proteins from a RIPA extract of cultured human myotubes. The beads were washed, digested with trypsin and the digest was analysed by nano-scale liquid chromatographic electrospray ionisation tandem mass spectrometry. A control pull-down with beads alone detected proteins that bind non-specifically to the beads and cytoskeletal proteins associated with early myogenesis (e.g. myosin-3 and nestin, as well as tubulin) were prominent among these. Although the method reliably identified over 500 proteins, all except about 150 were also pulled-down by beads alone. The bait protein, nesprin-1-alpha2, had the greatest number of significant and unique peptide sequences in both WT and STARdel pull-downs. Unfiltered mass spectrometry results for wild type, Star deletion and Control pull-downs are shown in Supplementary Table 1a–c respectively. Of the approximately 150 proteins pulled down by the recombinant proteins, only 2 proteins were pulled-down by wild-type nesprin-1-alpha2 and NOT pulled down by nesprin with the STAR deletion; kinesin heavy-chain (31 unique peptides) and kinesin light-chain (12 unique peptides) (Table 1).

**Table 1:** Mass spectrometry abridged results of Wild Type pull-down.

Accession number	No. of peptides	No. of significant peptides	No. of unique peptide sequences	No. of significant, unique peptide sequences	Description	
gi|28195677	566	512	45	41	nesprin-1 alpha 2 [Homo sapiens]	Found in Wt and Star deletion pull-downs. “Bait” protein
gi|459214634	45	16	31	13	KIF5B-RET(NM_020630)_K23;R12 fusion protein [Homo sapiens] (Kinesin 1, heavy chain)	Found only in Wt pull-down
gi|32452907	15	8	12	7	TPA_exp: kinesin light chain 1 O [Homo sapiens]	Found only in Wt pull-down

The protein with the most significant unique peptide sequences in both WT and STARdel pull-downs was nesprin-1-alpha2 itself (bait protein). When proteins that occur in STARdel and Control pull-downs were removed, only kinesin heavy chain and kinesin light chain were unique to the WT pull-down ( > 2 significant unique peptide sequences). See Supplementary Table 1 for full results. Note that the “bait” nesprin proteins for pull-downs lacked the KASH domain which binds SUN proteins.

Nesprin-2 peptides were not detected in any pull-downs; however, nesprin-2 is present at only very low levels in cultured myotubes^20^, so this study is not informative about possible interactions between nesprin-1 and nesprin-2.

**Figure 1:**
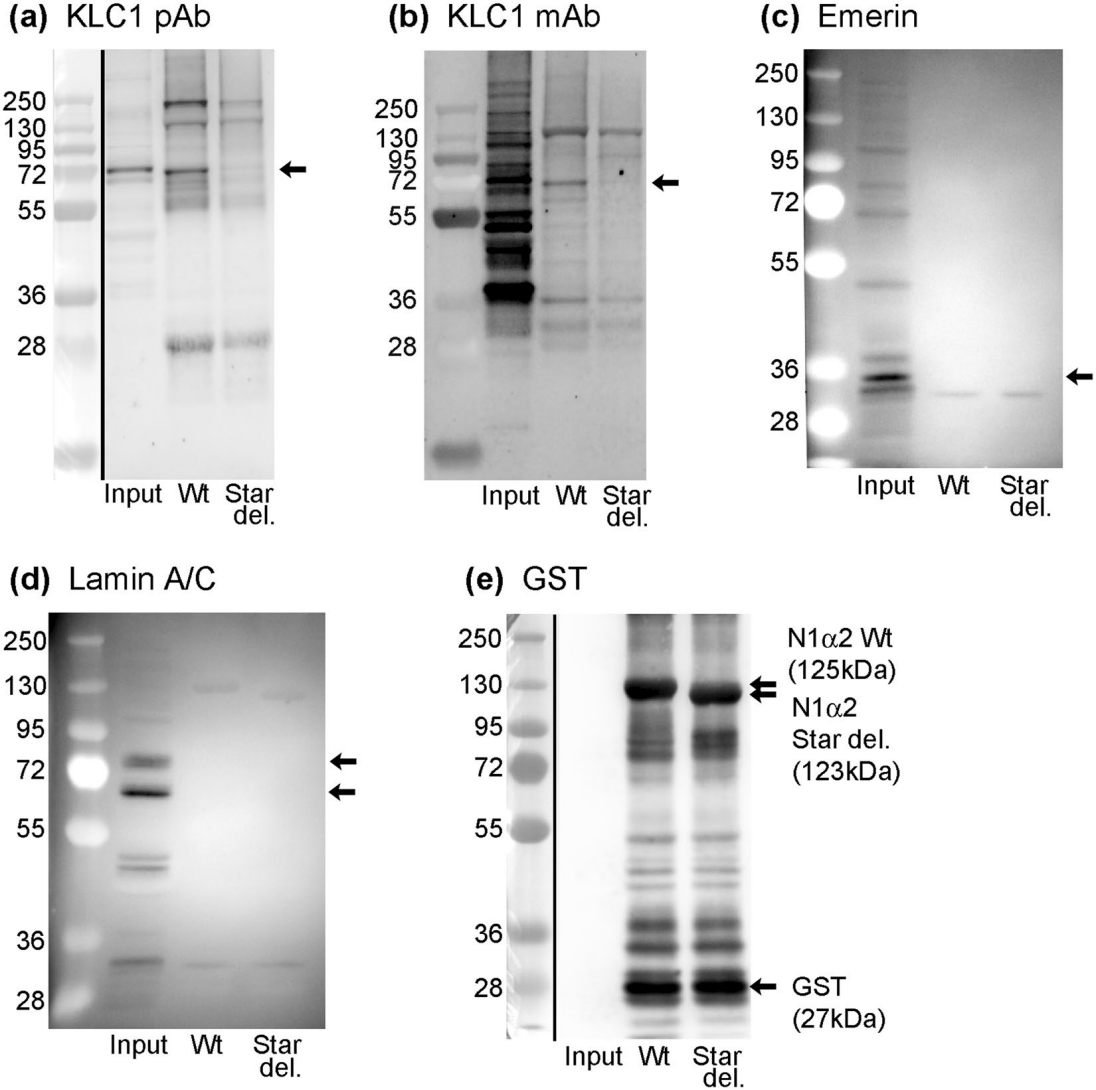
Nesprin-1-alpha2 pulls down kinesin light-chain from human myotube extracts, only if the STAR domain (with LEWD) is present. No pull-down of emerin or lamin A/C was detected. Extracts of human myotubes (Input) and the component of the myotube extract pulled-down by wild type N1α2 (Wt) or STAR deletion N1α2 (Star del) were probed with (**a**) KLC1 (rabbit pAb, GeneTex, GTX114510), (**b**) KLC1 (mouse mAb, Santa Cruz, sc-515792), (**c**) MANNEM1, emerin mAb, (**d**) MANNLAC1, Lamin A/C mAb or (**e**) mAb against GST tag as loading control. Whole blots are shown; black vertical lines separate the blue markers (taken with visible light) from the western blot (by chemiluminescence). In some blots (**b**–**d**), the markers were visible in the chemiluminescent exposure, so a separate visible-light exposure was not needed. Black arrows indicate bands of expected size.

We confirmed by western blot using two different antibodies that human kinesin light-chain (72 kDa) was in WT pull-downs but not in STARdel pull-downs (Fig. 1a, KLC1 pAb, Genetex; and Fig. 1b, KLC1 mAb Santa Cruz). The KLC1 mAb (Fig. 1b) and a KLC pAb (Abcam pAb ab187179, not shown), both cross-reacted with non-KLC proteins on western blots. Figure 1e is a loading control using antibody against the “bait” protein (GST-nesprin recombinant).

**Figure 2:**
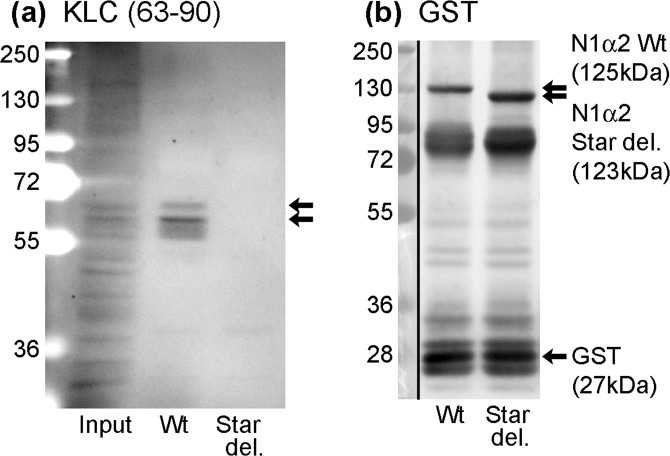
Nesprin-1-alpha2 pulls down kinesin light-chain from mouse C2C12 myotube extracts, only if the STAR domain (with LEWD) is present. Extracts of mouse C2C12myotubes (Input) and the component of the myotube extract pulled-down by wild type N1α2 (Wt) or STAR deletion N1α2 (Star del) were probed with (**a**) KLC (63–90) mAb or (**b**) mAb against GST tag as loading control. Whole blots are shown; black vertical lines separate the blue markers (taken with visible light) from the western blot (by chemiluminescence). In blot (**a**), the markers were visible in the chemiluminescent exposure, so a separate visible-light exposure was not needed. Black arrows indicate bands of expected size.

The same result was obtained using mouse C2C12 myotube extracts and a mAb that recognises mouse KLC1/2 (mAb 63–90; Molecular weight 62–65 kDa: Fig. 2a). Figure 2b is an anti-GST loading control.

The STAR domain^6^ lies within both of two overlapping regions of nesprin-1 that have been shown to bind lamin A/C^26^ and emerin^27^ respectively *in vitro*. Although both emerin and lamin A/C were easily detected in the myotube extract, they were not detected in any pull-downs, either by mass spectrometry (Supplementary Tables) or by western blot (Fig. 1c,d). This suggests that the *in vivo* interaction of nesprin-1-alpha2 with kinesin is much stronger than any interaction with either emerin or the lamin A/C present in the RIPA extracts. Because the recombinant nesprin used lacks the KASH domain, it would not be expected to pull down either SUN proteins or any lamin A/C and emerin attached to SUN proteins.

### Localization of nesprin-1-alpha2 in human skeletal myotubes

Using mAbs specific for nesprin-1-alpha2 and for nesprin-1-giant, as well as mAbs that recognize all nesprin-1 isoforms, we looked for co-localization with kinesin light-chain (KLC) in human myotube cultures. The mAb, N1G-ex130^24^, does not recognise nesprin-1-alpha2 and these are the only two isoforms significantly-expressed in muscle cultures^3^ so N1G-ex130 is effectively specific for nesprin-1-giant in myoblasts and myotubes.

**Figure 3:**
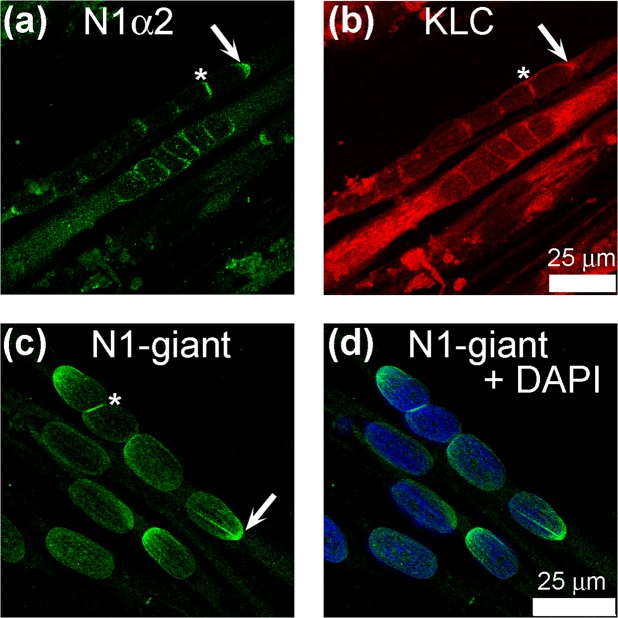
Both nesprin-1-alpha2 and nesprin-1-giant partially co-localize with kinesin light-chain at nuclear membranes in human myotube cultures. N1α2 (**a**), KLC (**b**) and Nesprin-1 giant (mAb N1G-Ex130) (**c**) were concentrated at the polar ends of myotubes (white arrows) and at the junctions where nuclei meet (asterix).

To localize KLC-1 in human myotubes, we selected a polyclonal Ab from Genetex which gave a single 72 kDa band on western blot (Fig. 1a). Antibodies from two other commercial sources showed cross-reactions with non-KLC-1 proteins on western blots. In myotubes containing linear assemblies of nuclei, KLC-1 was concentrated at the junctions between nuclei (Fig. 3a; asterix). The specific nesprin-1-alpha2 mAb enabled us to show that this protein co-localized with KLC-1 at the junctions (Fig. 3b; asterix). Both proteins were also found at the outer poles of some nuclear chains (Fig. 3a,b; arrows).

**Figure 4:**
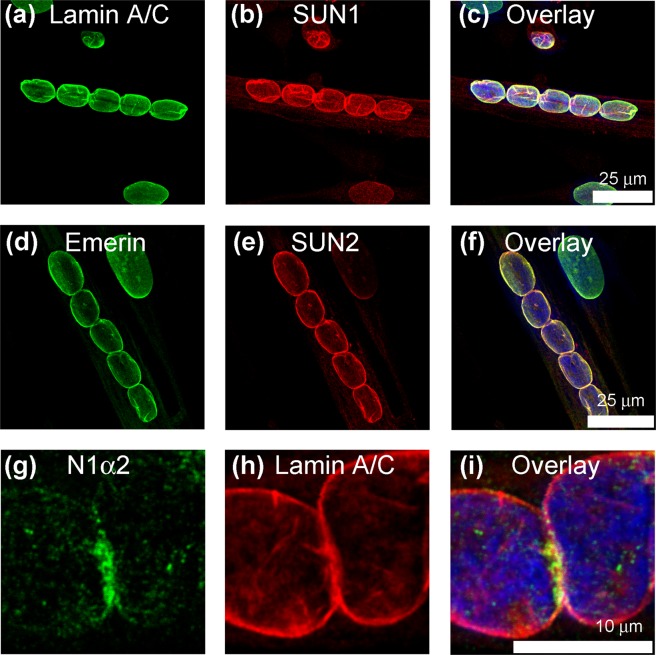
SUN1, SUN2, emerin and lamin A/C are more uniformly distributed on nuclear membranes in human myotube cultures. Lamin A/C (**a**,**h**) and inner nuclear membrane proteins SUN1 (**b**), emerin (**d**) and SUN2 (**e**) showed uniform distribution at the nuclear envelope which was often different from the uneven, more polar distribution of N1α2 (**g**).

The specific nesprin-1-giant mAb showed that nesprin-1-giant was also present at the junctions (asterix) but was also more evenly distributed around the nuclear rim (Fig. 3c). It was also found at one pole of some nuclei (Fig. 3c; arrow). Since both giant and short forms show this localization, one would expect that a mAb, such as our MANNES1A, which recognizes both forms, would show similar uneven staining of the nuclear rim. This is evident in photomicrographs we have previously published using MANNES1A (Fig. 4 in^24^), though we did not comment on the uneven localization at that time. Gimpel *et al*.^19^ also showed the location at nuclear junctions using a pan-nesprin-1 antibody, since the specific nesprin-1-alpha2 mAb was not available at that time.

In these short linear arrays of nuclei, emerin, lamin A/C and SUN proteins usually showed a uniform distribution on the nuclear membrane (Fig. 4), different from the distribution of nesprin-1 isoforms. Emerin and lamin A/C co-localised with each other, and usually with SUN1 and SUN2 (Fig. 4), as expected, but lamin A/C did not co-localize exactly with nesprin-1-alpha2 at nuclear junctions (Fig. 4g–i).

We checked the proportion of nuclei with staining concentrated at poles or junctions and found that 81 to 87% of concatenated myotube nuclei contained nesprin-1-alpha2, total nesprin-1 and kinesin localized at the poles or junctions, whereas the vast majority of nuclei had a uniform localization of SUN2 at the nuclear rim (Table 2). These studies were performed with immortalised, clonal human myoblast cell lines, but we also found that primary human myotube cultures gave essentially the same results (Supplementary Fig. S3).

**Table 2:** The proportion of myotube nuclei with specific proteins concentrated at poles or junctions of nuclei.

Protein (antibody)	Number of Nuclei			Percentage of nuclei with localization at poles or junctions
	Localization at poles or junctions of nuclei	Uniform localization at nuclear rim	Total number of nuclei	
Nesprin-1-alpha2 (1H2)	60	9	69	87%
Nesprin-1 (MANNES1A)	52	12	64	81%
Kinesin light-chain (Genetex pAb)	51	9	60	85%
SUN2 (pAb)	2	57	59	3%

For each antibody, a total of 6 coverslips were examined from a total of 3 experiments.

**Figure 5:**
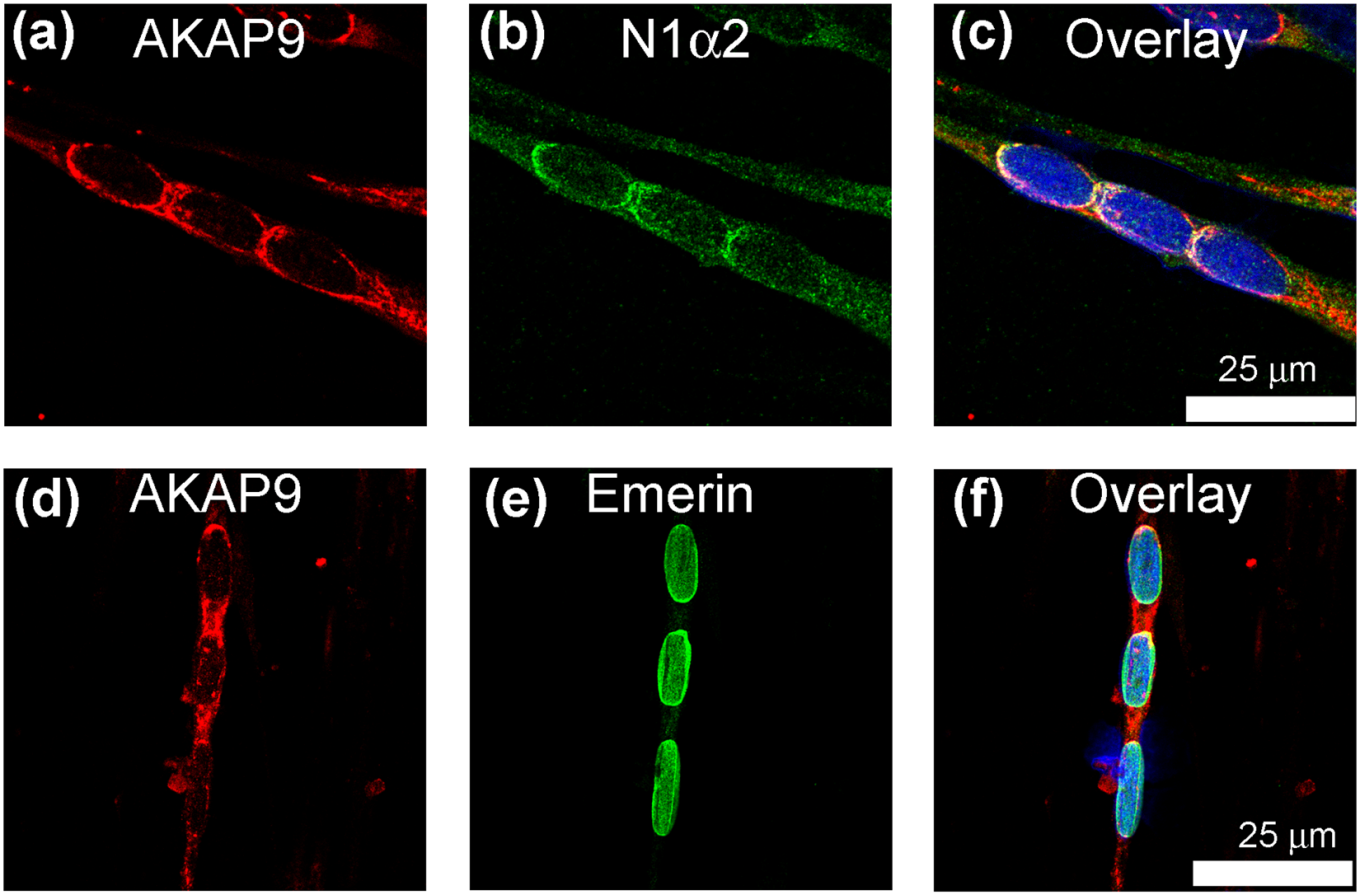
Partial co-localization of nesprin-1-alpha2 with the centrosomal protein AKAP9 in human myotube cultures. The centrosomal scaffolding protein AKAP9 was partially localized at the nuclear envelope (**a**) and also between nuclei (**d**). Emerin (**e**) had a uniform nuclear envelope distribution whereas nesprin-1-alpha2 (N1α2: **b**) showed better, though partial, co-localization with AKAP9.

As reported by Gimpel *et al*.^19^, AKAP9 (AKAP450), the centrosomal scaffolding protein, partially co-localizes with nesprin-1-alpha2 at the poles of nuclear chains, and between concatenated nuclei when these nuclei are close together (Fig. 5a–c). Unlike nesprin-1-alpha2, AKAP9 was also found between nuclei that are well-separated in the myotube (Fig. 5d). AKAP9 is found at several cellular locations, including the Golgi apparatus^28^.

### Localization of nesprin-1-alpha2 in adult rat skeletal and cardiac muscles

**Figure 6:**
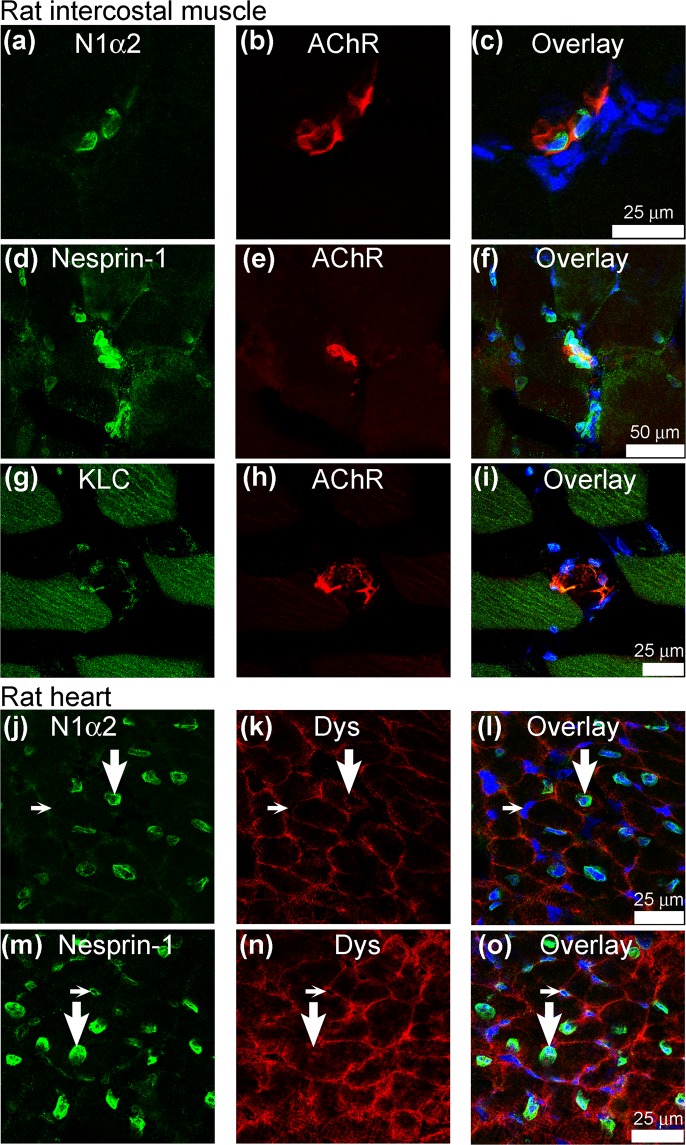
Nesprin-1-alpha2 is mainly restricted to neuromuscular junction nuclei in adult rat skeletal muscle and to cardiomyocyte nuclei in adult rat heart. Neuromuscular junctions were identified in transverse sections of rat intercostal muscle by labelling nicotinic acetylcholine receptors with alpha-bungarotoxin-TRITC (**b**,**e**,**h**). Nesprin-1-alpha2 (N1α2: **a**,**c**) was found almost-exclusively on nuclei associated with neuromuscular junctions, whereas total nesprin-1 (mAb MANNES1A) (**d**,**f**) was more widespread. Kinesin light-chain (**g**,**h**,**i**) was also found on many nuclei. Cardiomyocytes in sections of rat heart were identified by staining the sarcolemma for dystrophin (**k**,**n**). Examples of cardiomyocyte nuclei (large vertical arrows) and non-cardiomyocytes (small horizontal arrows). Nesprin-1-alpha2 (**j**,**l**) was predominantly on the nuclei of cardiomyocytes whereas total nesprin-1 (**m**,**o**) was also on nuclei of interstitial cells between cardiomyocytes.

In adult rat intercostal muscle, specific staining for nesprin-1-alpha2 was present on nuclei associated with neuromuscular junctions (as revealed by alpha-bungarotoxin binding), but not on non-junctional nuclei (Fig. 6a–c), most of which stain strongly for nesprin-2 and less strongly for nesprin-1-giant (as previously shown in^3^). Nesprin-1-giant (Fig. 6d–f) was found on most, but not all, non-junctional nuclei. Nesprin-1-negative nuclei at NMJs have previously been shown to include Schwann cell nuclei^29^. Kinesin light-chain was detectable on some NMJs (Fig. 6g–i). Further examples of nesprin-1-alpha2 and total nesprin-1 at neuromuscular junctions, in some cases with an additional dystrophin stain of the sarcolemma to outline the muscle fibres more clearly, are shown in Supplementary Fig. S4. In contrast, all rat cardiomyocyte nuclei were strongly stained for nesprin-1-alpha2 (Fig. 6j–l). Cardiomyocyte nuclei were identified by their location within dystrophin-bordered fibres (Fig. 6k,n). Other nuclei (in blood vessels, interstitial cells, etc.) in the heart section were not stained for nesprin-1-alpha2 (Fig. 6j–l), although nesprin-1-giant was present (Fig. 6m–o), suggesting a rather specific association of nesprin-1-alpha2 with cardiomyocyte nuclei.

Previous studies have shown that the giant and alpha2 isoforms are the main nesprin-1 components in cardiac tissue^3^. Rat tissues were used because of difficulties in obtaining good quality human heart sections or human muscle biopsy sections containing NMJs.

## Discussion

The special relationship between SYNE1 (nesprin-1) and myonuclei at NMJs was established in the original paper on SYNE1 by Apel *et al*.^1^. However, the existence of a larger SYNE1 gene product, nesprin-1-giant, was not known at that time and the antibody used by Apel *et al*. in^1^ would have detected all isoforms, so Fig. 6c is the first demonstration of a specific association of nesprin-1-alpha2 with nuclei at NMJs. Total nesprin-1 (mAb MANNES1A), was detected in most, though not all, nuclei on the sections.

The specialized nuclei associated with NMJs express high levels of mRNA for acetylcholine receptors and related proteins^30,31^. SYNE1 was found to be essential for the anchorage of these specialized myonuclei at NMJs, although NMJ function was unaffected when this anchorage was disrupted by over-expression of KASH sequences^32^. The mouse double knockout (DKO) of SUN1/SUN2 *in vivo* caused complete loss of nesprin-1 from the nuclear envelope of 18.5 day embryonic mouse intercostal muscles, while leaving lamin A/C and emerin at the inner nuclear membrane (INM) unaffected^33^. Strikingly, the association of nuclei with NMJ was almost completely lost in the DKO^33^. In the light of our present results, this is consistent with a role for SUN-anchored nesprin-1-alpha2 in localizing nuclei to NMJs. The association of kinesin with the NMJ nuclei (Fig. 6i) would be consistent with its involvement in their localization.

In contrast to adult skeletal muscle nuclei, all adult cardiomyocyte nuclei expressed nesprin-1-alpha2 at the nuclear envelope. This is not unexpected, since nesprin-1-alpha2 is required for the localization of mAKAP to the cardiac myocyte nuclear membrane^17^ and mAKAP has a central role in the assembly of signalling pathways that regulate cardiac growth and function^34^. The role of nesprin-1-alpha2 appears to be rather specific for cardiomyocytes since it was not detected in other cardiac cell types which do contain nesprin-1-giant.

In current models of nuclear movement during myogenesis^14,15^, SUN2 and nesprin-2-giant are involved in preparing the mononucleate myoblast for fusion to form myotubes. Nuclei migrate towards the centre of the myotube in a nesprin-independent manner. Within the myotube, nesprin-1 and kinesin engage with the microtubule/centrosome system to move and separate nuclei lengthwise. Nesprin-1-alpha2 is essential for reorganization of microtubules and kinesin to achieve this^13,18,19^. The microtubule-associated protein MAP7 is also involved in kinesin-associated myonuclear spreading^35^. We have shown here, using specific antibodies for the first time, that BOTH nesprin-1-giant and nesprin-1-alpha2 are concentrated with kinesin at nuclear poles/junctions in linear concatenations of nuclei in myotubes. We may possibly infer that nesprin-1-alpha2 is engaged at these sites in its likely function of separating nuclei lengthwise within the myotube, although it is worth noting that this function is imperfectly-achieved *in vitro*, since many nuclei in cultured human myotubes remain in contact with each other. It is not yet known whether each SUN trimer attaches to three molecules of a single nesprin isoform or to mixtures of nesprin isoforms that might even include both nesprin-1 and nesprin-2 molecules. However, the observation that nesprin-1-alpha2 does not completely co-localize with nesprin-1-giant on the whole NE suggests that some mechanism for non-random association of nesprin-1 isoforms with SUN trimers may exist.

In our proteomics study, the evidence for specific binding of the nesprin-1 STAR domain (containing the LEWD motif) to kinesins was striking and we found no evidence for binding of nesprin-1-alpha2 to emerin or lamins under the same experimental conditions. Kinesin heavy and light chains were the only proteins pulled-down from human myotube extracts by a nesprin-1-alpha2 bait protein but not also pulled-down by the same bait lacking the STAR domain. Kinesin-binding is likely the only function for the highly-conserved STAR domain, which contains the LEWD motif^9^ and is shared by nesprins 1 and 2^6^. SUN proteins were not pulled down in this study because a KASH-less form of nesprin-1-alpha2 was used. Our observation that neither emerin nor lamin A/C was detected in pull-downs by nesprin-1-alpha2 does not prove a lack of direct interaction, but it has led us to re-evaluate the evidence.

It was once a reasonable and popular hypothesis that giant forms of nesprins have functions at the outer nuclear membrane (ONM) while the shortest forms are at the INM (reviewed in^25^). This was largely based on evidence that nesprins interact directly with lamin A/C and emerin, both of which are associated with the INM^26,27^. The hypothesis was encouraged by the presence of a possible interrupted LEM domain in nesprin-1-alpha for interaction with chromatin via BAF^26^, although BAF binding was not confirmed^26,36^. The present results, with those of others^7–9,17–19^, are important in showing a function for nesprin-1-alpha2 at the ONM of striated muscle nuclei, so the question has become whether nesprin-1-alpha2 has any additional function at the INM. Although there is good evidence that recombinant nesprins can bind with high affinity to both emerin and lamin A/C *in vitro*^26,27,36^, these interactions may not occur *in vivo* if nesprin-1-alpha2 does not normally come into contact with the nuclear lamina (because it is in the ONM). Once you make cell extracts for pull-downs, proteins attached to the ONM and INM are no longer separated; non-physiological interactions can then occur and this limits the value of simple pull-downs in determining protein localization.

Current light microscopy cannot resolve whether nesprins are at the ONM only or at both ONM and INM. Two immuno-EM studies of nesprin-2 in skeletal muscle^36,37^ were ambiguous in suggesting that both giant and short forms may be present at both INM and ONM. However, the technical difficulties are considerable; many epitopes for mAbs are lost after harsh fixation methods required for EM and this may explain why few EM studies have been attempted. The interaction of the SUN protein trimer with BOTH lamin A/C at the INM AND with the nesprin KASH domain inside the lumen is clearly oriented for placing nesprins at the ONM^7,8^; the well-characterized “SUN domain- KASH domain” interaction would not direct nesprin-1-alpha2 to the INM. There is *in vitro* evidence using recombinant proteins for the potential localization of a nesprin-2-beta short form at the INM by a non-KASH interaction between the nucleoplasmic domain of SUN1 and the spectrin-repeat region of nesprin-2-beta^38^ but similar interactions could not be demonstrated for nesprin-1 short forms in the same study^38^ and nesprin-2-beta is not endogenously expressed in striated muscle^3^. Splicing isoforms of nesprin-1 which lack the KASH domain do exist, but the last exon of SYNE1 encodes both KASH and transmembrane domains of nesprin, so any splicing isoform lacking KASH will also lack the TM domain. These splicing isoforms of nesprins lacking both TM and KASH regions may enter nuclei, if they have a functional NLS, but there is evidence that they locate to the cytoplasm^39^ or the nucleoplasm^3^, rather than co-localizing with lamins at the inner nuclear membrane.

The continued presence of emerin and lamin A/C in SUN1/SUN2 double-knockout nuclei was not sufficient to retain nesprin-1 at the nuclear envelope^33^, and this would seem to be strong support for localization of most, if not all, nesprin-1 at the ONM by SUN proteins. To understand the *in vivo* location of nesprin-1-alpha2, it is necessary to study the tissue in which nesprin-1-alpha2 is normally expressed, striated muscle^1–3^. Non-muscle cells and tissues usually express only nesprin-1-giant^3^, so *in vivo* studies using null mutants and siRNA knockdowns do not help with the problem of nesprin-1-alpha2 localization, but they do raise other problems. Emerin-null human fibroblasts *in vitro* retain lamin A/C and both SUN proteins at the nuclear envelope and also retain nesprin-1-giant (predictably), but they sometimes^37^ or usually^20^ fail to retain nesprin-2-giant, which is rather difficult to explain. Furthermore, emerin-null keratinocytes (which lack nesprin-1) do retain nesprin-2-giant at the nuclear envelope in all cells^20^. In lamin A/C knockout mouse embryonic fibroblasts (MEFs)^40^, both SUN proteins (SUN1 in all cells, SUN2 in some cells^41^) remained at the nuclear envelope while emerin and both nesprins (the giant isoforms) were displaced to the ER in all cells^20,36,37^; this suggests that SUN proteins unanchored to the lamina are unable to retain giant nesprins at the ONM, for reasons that are not yet clear. It would be dangerous to interpret these results as evidence for direct lamin A/C interaction with nesprins at the INM, in view of the strong evidence that most, if not all, giant forms are located at the ONM.

We conclude that the principal function of nesprin-1-alpha2 at the nuclear rim of muscle cell nuclei is at the ONM rather than the INM. Without a more reliable method for direct observation than EM, it is not possible to exclude some additional function for nesprin-1-alpha2 at the INM, but more evidence is needed before it becomes essential to invoke an INM function.

## Materials and Methods

### Ethical approvals

This study was approved by the RJAH Orthopaedic Hospital Research Committee. All human tissue studies complied with the UK Human Tissue Act (2006). Written informed consent in accordance with European recommendations and French legislation was obtained for muscle biopsies for isolation and immortalisation of myoblasts^42^. Protocols approved by the MRC Centre for Neuromuscular Disease Biobank, London, were followed when obtaining informed written consent for other muscle biopsies. All participants under 16 years old provided written informed consents from parents or legal guardians.

### Nesprin-1-alpha2 “Bait” proteins

The expression plasmid pGEX-4T1/N1α2 SR1-5^10^ produces aa1-842 of the 978aa nesprin-1-alpha2 with GST attached to the amino-terminus. The expressed GST-fusion protein lacks the final spectrin repeat and both KASH and transmembrane domains.

Site-directed mutagenesis (QuikChange II XL; Cat No: 200521; Agilent Technologies, Cheadle, UK) using the following sense (5′-ccggagcgagcgggactatgacctcag-3′) and antisense (5′-ctgaggtcatagtcccgctcgctccgg-3′) primers (Life Technologies, Warrington UK) was used to delete 54 nucleotides corresponding to the entire STAR domain. Successful deletion was confirmed by sequencing (Supplementary Fig. S2). Recombinant N1-α2 wild type (Wt) and STARdel proteins, with GST tags, were expressed in E.coli and extracted by sonication in TNE, followed by sonication in increasing concentrations of urea. A Coomassie Blue-stained gel showed that most of the recombinant proteins were extracted in the TNE fraction and this was used in subsequent work.

### Muscle cell culture

A previously-described method was followed^24^. Clonal myoblast cell lines were immortalized by transduction with human telomerase reverse transcriptase (hTERT) and cyclin-dependent kinase-4 (Cdk4) containing retroviral vectors, at the Institut de Myologie, Paris, as described previously^42^. Immortalized myoblasts from a 25 year old donor^24,42^ were cultured in skeletal cell growth medium (Cat No: C-23060; PromoCell GmbH, Heidelberg, Germany) containing supplement mix (Cat No: C-39365; PromoCell) with 20% Fetal Bovine Serum (Cat No: 10270; Gibco; ThermoFisher Scientific, Paisley, UK). Differentiation was induced at 80% confluency by washing the adherent myoblasts in medium lacking serum and then culturing in DMEM (Cat No: 31966-021; Gibco; ThermoFisher Scientific) supplemented with Insulin (1721nM), Transferrin (68.7 nM), Selenium (38.7 nM) (ITS-X; Cat No: 51500-056; Gibco; ThermoFisher Scientific) and Penicillin-Streptomycin (Cat No: DE17-603E; Lonza; Verviers, Belgium). After a further 4 days of cell culture, over 80% of the cells had fused into myotubes. Cells cultured for pull-down experiments were extracted in RIPA buffer (1% NP-40; 0.25% deoxycholic acid; 1 mM EDTA; 150 mM NaCl; 50 mM Tris-HCl, pH 7.4), in the presence of protease inhibitors (Sigma P8340 plus 1 mM PMSF), by sonication followed by centrifugation.

### Pull-down and mass spectrometry

Glutathione high capacity magnetic agarose beads (Cat No: G0924; Sigma) were loaded with GST-tagged “Bait” proteins (N1-α2 Wt, N1-α2 Star del, or No Bait control) and subsequently incubated with “Prey” (cultured myotube extract in RIPA buffer). Captured proteins from the human myotube extract were digested by addition of trypsin 1%v/v and incubation at 37 °C overnight. One third of the sample was diluted in loading buffer (98% water 2% acetonitrile 0.05% TFA) and analysed on the nanoLC MSMS as follows:

A nanoLC as-2 autosampler and nanoLC Ultra 2D plus loading pump (Eksigent) were used to load peptides onto an Acclaim PepMap 100 C18 trap in in 98% water, 2% acetonitrile, 0.05% TFA. The trap was washed for 20 mins to waste before switching in line with an Acclaim PepMap RSLC C18 column (ThermoFisher Scientific). Peptides were subsequently eluted from the column with a gradient of increasing acetonitrile containing 0.1% formic acid (i.e. 2–20% acetonitrile for 90 min, 20–40% for a further 30 min, followed by 98% acetonitrile to clean the column, before re-equilibration to 2% acetonitrile). The eluate from the column was sprayed into a TripleTOF 5600 electrospray tandem mass spectrometer (ABSciex) and analysed in Information Dependent Acquisition (IDA) mode by performing 120 msec of MS followed by 80 msec MS/MS analyses on the 20 most intense peaks seen by MS. An MS/MS data file was generated using the ‘Create mgf file’ script in PeakView (Sciex) for analysis using the Mascot search algorithm (Matrix Science). Proteins were identified by searching the file against the NCBInr database (Aug 2016), with the following search settings: species set to Homo sapiens (331,464 sequences), trypsin as the cleavage enzyme, methionine oxidation as a variable modification, peptide mass tolerance of 20ppm, and an MS/MS mass tolerance of ±0.1 Da. This analysis was used to identify medium-to-high abundance proteins that interacted with wild-type, but not with mutant, nesprin-1-α2.

### SDS-polyacrylamide gel electrophoresis and Western blotting

Methods were modified from those previously described^3,24^. Samples were mixed with SDS buffer (125 mM Tris pH 6.8; 2% SDS; 5% 2-beta mercaptoethanol; 5% glycerol; with bromophenol blue). After boiling, samples were subjected to SDS-PAGE using 10% polyacrylamide gels and transferred to nitrocellulose membranes (Protan BA85, Whatman). After blocking non-specific sites with 5% skimmed milk protein, membranes were incubated with primary antibody: Rabbit pAb KLC1 [N2C2] (GeneTex, Cat No: GTX114510) or rabbit pAb KLC1 (Abcam, Cat No: ab187179), or the following mouse mAbs: KLC (H7) (Santa Cruz, Cat No: sc-515792), KLC (63–90) (Prof. Scott Brady^43^, University of Illinois at Chicago), or MANEM1 5D10 against emerin^44^, MANLAC1 4A7 against Lamin A/C^45^, or mAb GST 17A10 (raised against recombinant GST). This was followed by washing and incubation with either peroxidase-labelled goat anti-rabbit immunoglobulins (Dako, Denmark, Cat No: P0448) or peroxidase-labelled rabbit anti-mouse immunoglobulins (Dako, Cat No: P0260) (1/1000). Antibody reacting bands were detected with SuperSignal West Femto chemiluminescent detection system (ThermoFisher Scientific, Cat No: 34094) and ChemiDoc Touch imaging system (BioRad Ltd.).

### Immunofluorescence microscopy

Methods were modified from those previously described^3,24^. Immunohistochemistry was performed on cells cultured on coverslips, fixed with 50:50 acetone-methanol and washed with PBS, or on 10 μm unfixed cryostat sections of intercostal muscle and heart ventricle from an adult rat. The following monoclonal antibodies in culture supernatants were diluted in PBS and incubated on the specimen for 1 hour: N1alpha2-1H2 (Nesprin-1-alpha2)^24^; N1G-Ex130 (Nesprin-1 giant)^24^; MANNES1A (total Nesprin-1)^20^; MANNEM1 (Emerin)^44^ or MANNLAC1 (Lamin A/C)^45^. Specimens were then washed and incubated with rabbit polyclonal antibody: KLC1 [N2C2) (GeneTex); SUN1 (anti-UNC84A, Cat No: HPA008346, Sigma); SUN2 (Dr. Sue Shackleton, University of Leicester); AKAP9 (Cat No: HPA026109, Sigma); Lamin A/C pAb or Dystrophin (rabbit pAb raised against C-term and mid regional peptides of dystrophin). To label neuromuscular junctions, alpha-bungarotoxin tetramethyl rhodamine (Cat No: T0195, Sigma) was diluted to 300 nM and incubated on the sections to bind nicotinic acetylcholine receptors. Following washing, incubation was continued with 5 µg/ml goat anti-mouse ALEXA 488 (Cat No: A11029, Molecular Probes, Eugene, Oregon, USA) and goat anti-rabbit ALEXA 546 (Cat No: A11010, Molecular Probes) secondary antibodies in PBS containing 1% horse serum, 1% fetal bovine serum and 0.1% BSA, for 1 hour. DAPI (diamidinophenylindole at 200 ng/ml) was added for the final 10 minutes of incubation to counterstain nuclei before mounting in Hydromount (Merck). Sequential confocal scans were performed with a Leica TCS SP5 spectral confocal microscope (Leica Microsystems, Milton Keynes, UK).

## Supplementary information

Figures S1-S4

Dataset 1
