# Supplementary material for: Nesprin-1-alpha2 associates with kinesin at myotube outer nuclear membranes, but is restricted to neuromuscular junction nuclei in adult muscle
Source: Sci Rep. 2019 Oct 2;9:14202. doi: 10.1038/s41598-019-50728-6 (PMC6775114; doi:10.1038/s41598-019-50728-6)
Supplement: Supplementary file 1 — Figures S1-S4 [file 41598_2019_50728_MOESM1_ESM.pdf]

Nesprin-1-alpha2 associates with kinesin at myotube outer nuclear membranes, but is restricted to neuromuscular junction nuclei in adult muscle

Ian Holt, Heidi R. Fuller, Le Thanh Lam, Caroline A. Sewry, Sally L. Shirran, Qiuping Zhang, Catherine M. Shanahan and Glenn E. Morris

Supplementary Figures

Figure S1

Recombinant protein expression

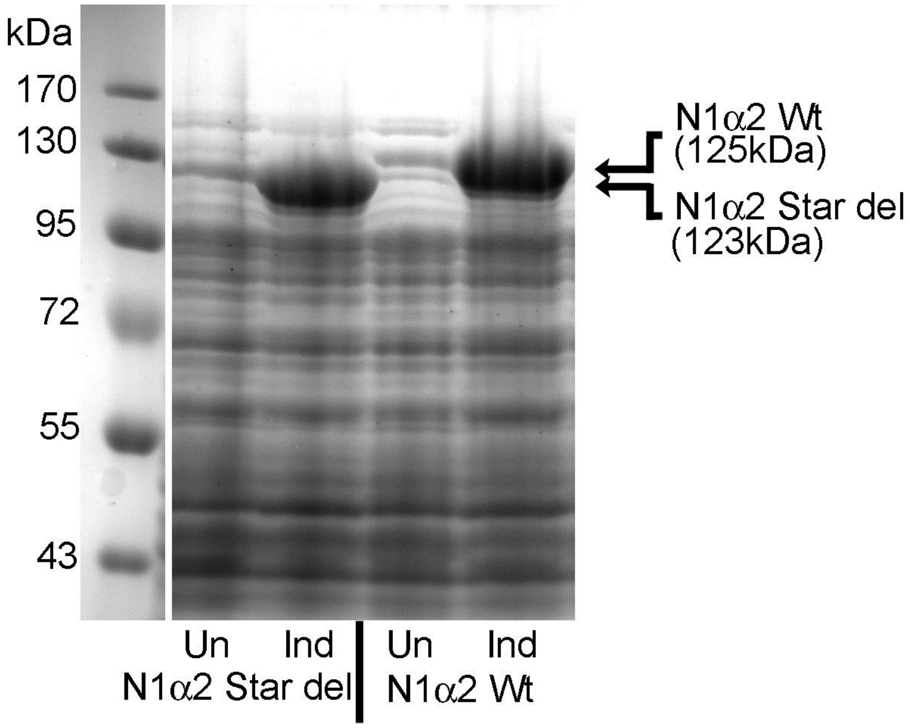

Stained gel showing extractions of uninduced (Un) and induced (Ind) bacterial cultures. Bands of recombinant Nesprin-1-alpha2 protein (Wild type or Star deletion) of expected size were present in induced cultures. The size marker lane has been sliced vertically from the same gel and juxtaposed next to the lanes of interest.

Figure S2

Sequence chromatograms

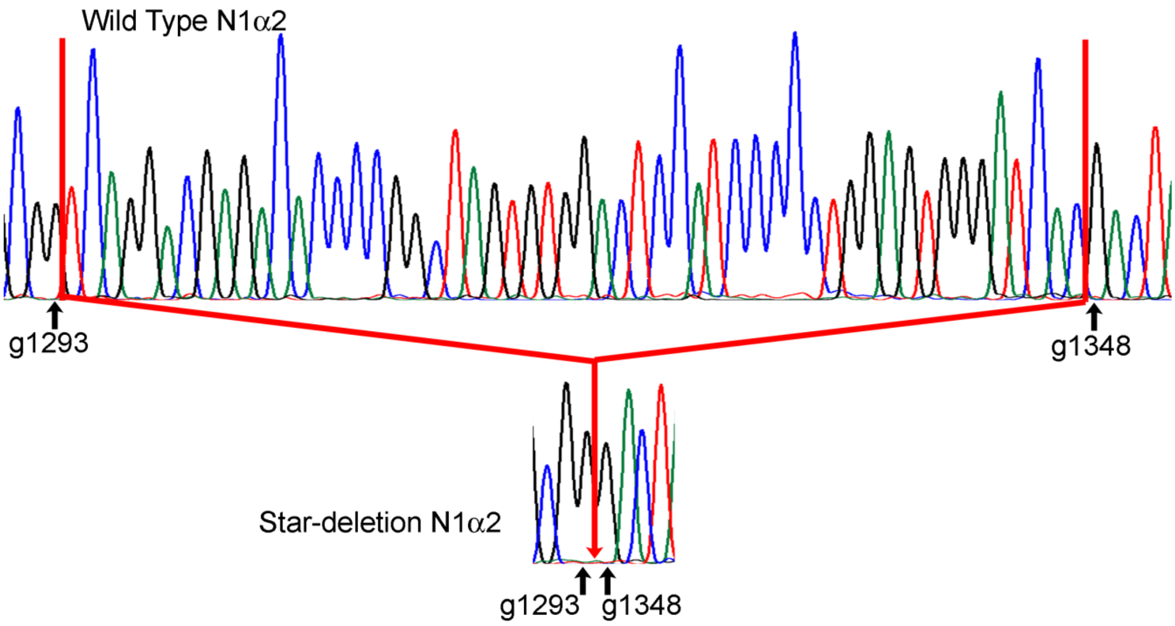

Sequencing of Wild type N1a2 (upper) and Star-deletion N1a2 (lower) confirmed the absence of nucleotides 1294 to 1347 from the star deletion cDNA. The two sequences were otherwise identical.

Figure S3

Primary human myotube cultures

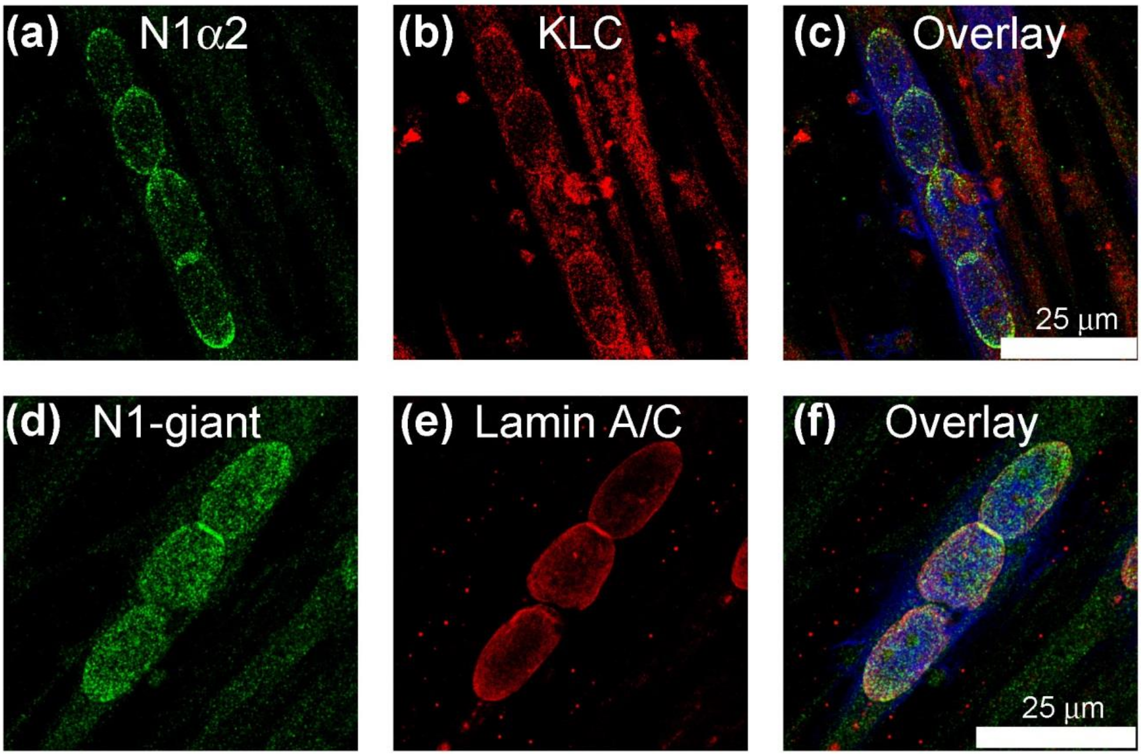

Immunofluorescent staining of non-immortalised human myotube cultures showed similar localisation patterns as seen with immortalised cell lines (see figs. 3, 4 and 5).

Figure S4

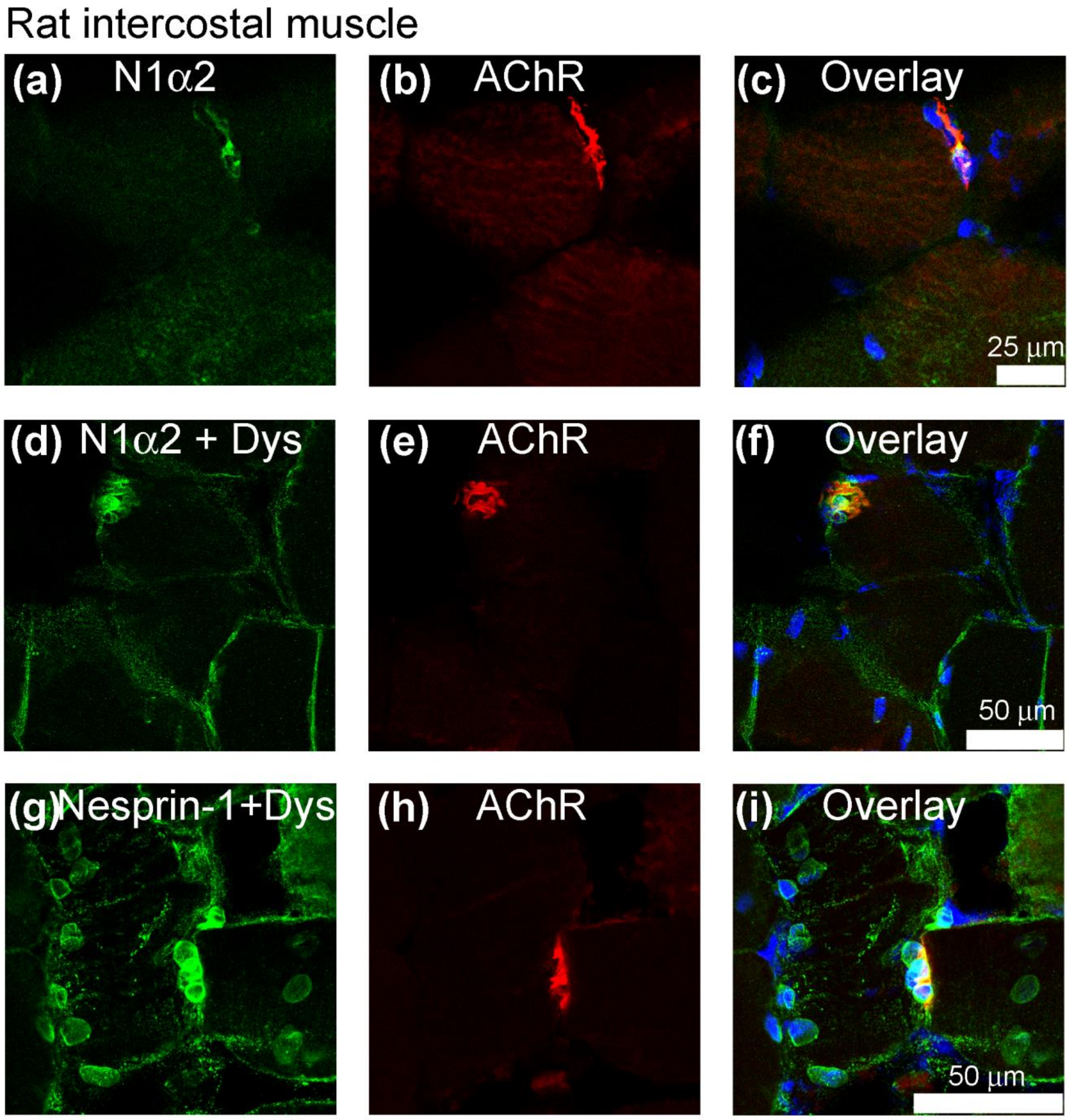

Immunofluorescent staining to show that nesprin-1-alpha2 (a to f) is mainly restricted to neuromuscular junction nuclei in transverse sections of adult rat intercostal muscle, whereas total nesprin-1 (g to i) was found on many nuclei. Neuromuscular junctions were identified using alpha-bungarotoxin-TRITC (red) (b,e,h). To label the sarcolemma, a dystrophin mAb (MANDYS1) was included with the nesprin-1 mAb (d and g) and both mAbs were detected by the same ALEXA-488 labelled secondary antibody (green).
